# Predictive value of sperm deoxyribonucleic acid (DNA) fragmentation index in male infertility

**DOI:** 10.1186/s12610-016-0046-3

**Published:** 2017-02-21

**Authors:** Budi Wiweko, Pramety Utami

**Affiliations:** Department of Obstetrics and Gynecology, Faculty of Medicine, Universitas Indonesia, Dr. Cipto Mangunkusumo General Hospital, Jl. Diponegoro no. 71, Central Jakarta, Indonesia

**Keywords:** Cut-off point, Male infertility, Halosperm**®**, Semen analysis, Sperm chromatin dispersion (SCD), Sperm DNA fragmentation index, valeur seuil, infécondité masculine, Halosperm^TM^, analyse de sperme, dispersion de la chromatine spermatozoïdaire (SCD), indice de fragmentation de l’ADN spermatozoïdaire

## Abstract

**Background:**

Recently, damage to the sperm DNA has been studied as it is associated with reduced fertilization rates, embryo quality, and pregnancy rates, also higher rates of spontaneous miscarriage.

**Objective:**

To develop a diagnostic method in predicting male infertility.

**Material and Methods:**

The design of this study is cross-sectional. Data were retrieved from medical records of Yasmin IVF Clinic Dr. Cipto Mangunkusumo General Hospital and Daya Medika Infertility Clinic from January to December 2015. Subjects were selected by consecutive sampling and divided into two groups: infertile and fertile. Sperm deoxyribonucleic acid fragmentation index (DFI) was determined by sperm chromatin dispersion (SCD) method using Halosperm® Kit.

**Results:**

There were 114 subjects (36 fertile and 78 infertile) selected into this study. We found no significant difference in the age between both of groups. The median value of sperm DFI in infertile group was significantly higher, 29.95 (26.6–34.3)%, compared to 19.90 (15.6–24.4)% of the fertile group, with *p* < 0.001. Area Under Curve (AUC) of sperm DFI, 0.862 (95% CI 0.783, 0.941), was higher than concentration (AUC 0.744; 95% CI 0.657, 0.831), motility (AUC 0.668; 95% CI 0.572, 0.765), and morphology (AUC 0.718; 95% CI 0.697, 0.864) of the semen analysis. At the cut-off point of 26.1%, the sperm DFI had sensitivity of 80.8% (95% CI; 70.0, 88.5), specificity of 86.1% (95% CI; 69.7, 94.8), positive predictive value (PPV) of 92.6% (95% CI; 83.0, 97.3), negative predictive value (NPV) of 67.4% (95% CI; 51.9, 80.0), positive likelihood ratio (PLR) of 12.6 (95% CI; 5.4, 29.4), and negative likelihood ratio (NLR) of 0.48 (95% CI 0.31, 0.75). Sperm DFI of ≥26.1% had prevalence ratio of 2.84 (95% CI 1.86, 4.33) for the occurrence of male infertility.

**Conclusion:**

There was significant difference between the median value of sperm DFI of infertile men and fertile men. Compared to semen analysis, sperm DFI at cut-off point of 26.1% has a higher diagnostic value (AUC).

## Résumé

**Objectif:**

Développer une méthode diagnostique e prédiction de l’infécondité masculine.

**Matériel et Méthodes:**

il s’agit d’une étude transversale. Les données ont été extraites des dossiers médicaux de la Yasmin IVF Clinic Dr Cipto Mangunkusumo General Hospital et de la Daya.

Medika Infertility Clinic, de janvier à décembre 2015. Les sujets ont été sélectionnés par échantillonnage consécutif et divisés en deux groupes : infécond et fécond. L’indice de fragmentation de l’acide désoxyribonucléique (DFI) a été déterminé par la méthode de la dispersion de la chromatine spermatozoïdaire (SCD) en utilisant le kit Halosperm™.

**Résultats:**

Cent quatorze sujets ont été sélectionnés dans cette étude : 36 féconds et 78 inféconds. Aucune différence significative n’a été retrouvée pour l’âge entre les deux groupes. La valeur médiane du DFI spermatozoïdaire dans le groupe infécond était significativement plus élevée, 29.9% (IQ1-IQ3 = 26.6–34.3%), que celle du groupe fécond, 19,90% (15.6–24.4%; *P* < 0.001). L’aire sous la courbe (AUC) du DFI spermatozoïdaire, 0.862 (95% CI 0.783, 0.941), était plus grande que celle de la concentration (AUC 0.744; 95% CI 0.657, 0.831), mobilité (AUC 0.668; 95% CI 0.572, 0.765), et morphologie (AUC 0.718; 95% CI 0.697, 0.864) des spermatozoïdes retrouvées à l’analyse du sperme. Pour une valeur seuil de 26,1%, le DFI spermatozoïdaire a une sensibilité de 80.8% (95% CI; 70.0, 88.5%), une spécificité de 86.1% (95% CI; 69.7, 94.8%), une valeur prédictive positive (PPV) de 92.6% (95% CI; 83.0, 97.3%), une valeur prédictive négative (NPV) de 67,4% (95% CI; 51.9, 80.0%), un ratio de probabilité positive (PLR) de 12,6 (95% CI; 5.4, 29.4), et un ratio de probabilité négative (NLR) de 0.48 (95% CI 0.31, 0.75). Un DFI spermatozoïdaire ≥ 26,1% a un ratio de prévalence de 2,84 (95% CI 1.86, 4.33) pour la survenue d’une infécondité masculine.

**Conclusion:**

La valeur médiane du DFI spermatozoïdaire diffère significativement entre les féconds et les inféconds. Par comparaison aux paramètres spermatiques de l’analyse du sperme, un DFI spermatozoïdaire à une valeur seuil de 26,1% possède une plus grande valeur diagnostique (AUC).

## Background

The incidence of infertility ranges from 10 to 20% in the world. This figure is similar with the data in Indonesia, where there are 39.8 million couples of childbearing age with 3.98 million (10–15%) experiencing infertility [1]. The success of a pregnancy is influenced by both men and women. Of all infertility cases, nearly 50% are due to male factor of infertility, either as a single factor or as a combination with female factor [2]. A study conducted by Wiweko, et al., found that male factor had a role in 43.9% of 266 infertile couples in Indonesia [3]. Male infertility is determined by the quality of the spermatozoa, which affects the ability for fertilization. In infertility cases, semen analysis that evaluates sperm concentration, motility, and morphology is done as a standard diagnostic tool to assess sperm quality. Damage to the sperm’s DNA has been studied lately as it is associated with reduced fertilization rates, embryo quality, and pregnancy rates, as well as higher rates of spontaneous miscarriage [4, 5]. A recent study, which studied the relationship between sperm DNA fragmentation in 203 couples underwent In Vitro Fertilization (IVF) and 136 couples underwent Intracytoplasmic Sperm Injection (ICSI), found that following the IVF, couples with <25% sperm DFI had a live-birth rate of 33% while couples with >50% DFI had only 13% birth rate. On the contrary, following the ICSI, there was no significant difference between both groups [6]. Live-birth rate increased significantly in couples with low sperm DFI compared to those with high sperm DFI (RR 1.17, 95% CI 1.07, 1.28; *p* = 0.0005) in a systematic review and meta-analysis [7]. Therefore, undetected sperm DNA damage could affect the outcome of Assisted Reproductive Technology (ART) and also potentially become a stumbling block in ART making the success rate relatively low, about 20–40% [3]. It also indicates that semen analysis is not optimal to detect male infertility, so that there is an opportunity to use a new predictive factor as a better diagnostic tool. Acridine orange test, Terminal deoxynucleotidyl transferase dUTP nick-end labeling (TUNEL) assay, Comet assay, sperm chromatin structure assay (SCSA), and sperm chromatin dispersion (SCD) are some methods commonly used to identify sperm DNA fragmentation. TUNEL assay is considered as the most superior method of all other methods, yet it needs more advanced equipment and high cost [8]. Chohan (2006) concluded that SCD had positive correlation with TUNEL assay results that they had similar predictive values, only SCD was a simpler and a low-cost method [9]. Another study by Zhang (2010) found that SCD was more sensitive than TUNEL assay to detect sperm with DNA fragmentation [10]. This study used Halosperm® Kit, which is a more accurate and efficient form of SCD method [11].

## Methods

### Patients

This study used a cross-sectional design. The data was retrieved from patient’s medical records of two infertility clinics in Jakarta (Yasmin IVF Clinic of Dr. Cipto Mangunkusumo General Hospital and Daya Medika Infertility Clinic) since January 2015 to December 2015. In this study, there were two groups of male subjects, infertile and fertile. The infertile group was obtained from couples who had undergone infertility workup with abnormal semen analysis (except azoospermia) and no cause of infertility in the female partner, while the fertile group was obtained from men proven to have a living child, taken from secondary infertile couples with abnormalities only in the female factor. Informed consent was obtained before all subjects recruited into this study. Ethical clearance was approved by Ethical Committee Faculty of Medicine Universitas Indonesia. From the sample size calculation, it was determined a minimum sample size of 110 subjects. Potential confounding factors, such as smoking, scrotal heat-exposure jobs, history of varicocele, cancer, genitourinary tract and gland infection, were obtained from the medical history and the examination results cited in the medical records. The last five conditions mentioned above were excluded from this study. Exclusion also applied for azoospermia as sperm DFI determination could not be performed.

### Semen analysis

After 7 days of abstinence, a complete sample of neat ejaculate produced by masturbation in a room in the laboratory was collected into a sterile container. In the first 30 min after the collection, sperm concentration was determined by using counting chamber technique, while wet preparation for sperm motility and eosin-stained for vitality determination were prepared. The slides were examined with phase-contrast optics at x400 magnification and only morphologically normal spermatozoa were assessed. An air-dried, fixed, and Giemsa-stained preparation was made for sperm morphology determination by using bright-field optics. The prominent semen analysis parameters in this study were sperm concentration, motility, and morphology by referring to the World Health Organization (WHO) standards 2010 [12]. The sperm DFI was determined by SCD test using Halosperm® Kit. Both determinations, semen analysis and sperm DFI, were carried out by the same expert.

### Statistical analysis

Statistical analysis was performed using SPSS version 23. Descriptive method was used to determine the distribution of the demographic profiles and the risk factors. Logistic regression was done to assess the smoking habit as the potential confounding factor. Due to the normality test of Kolmogorov-Smirnov showed abnormal data distribution, the Mann Whitney test was used to determine the association of semen parameters (concentration, motility, or morphology) and sperm DFI with male infertility. Categorization of sperm DFI was based on the cut-off point obtained from the Receiver Operating Characteristic (ROC) curve of sperm DFI.

## Results

From the medical records, it was obtained a total of 114 subjects fulfilling the study criteria. Subjects were divided into two groups: 78 of infertile group and 36 of fertile group. The median and range of age were 34 (32–39) years old in infertile group and 37 (35–41) years old in fertile group. Age did no significantly differ between fertile and infertile groups (*p* = 0.310). Smoking habit was not considered as confounding factor in this study based on logistic regression result (*p* = 0.401). Infertile group had a significantly higher median value of sperm DFI 29.9 (26.6–34.3)% compared to fertile group 19.9 (15.6–24.4)% with *p* < 0.001. With the same abstinence/delay in both groups, the values of sperm concentration, total motility, progressive motility, morphology, and total sperm count were significantly lower in the infertile group compared to the fertile group, while semen volume did not differ between these two groups (Table 1).

**Table 1:** Comparison of semen parameters and sperm DFI between fertile and infertile groups

	Fertile (*N* = 36)	Infertile (*N* = 78)	p
Semen analysis ^a^			
Abstinence (days)	7	7	
Vol (ml)	3.8 (2.8–3.6)	3.0 (2.1–3.4)	0.261
Con (x10^6^/ml)	49.0 (27.3–96.3)	15.3 (7.8–57.5)	<0.001
PR + NP (%)	53.9 (45.1–64.9)	40.6 (24.7–60.1)	<0.001
PR (%)	46.7 (37.3–60.8)	33.4 (26.2–34.1)	<0.001
Morph (%)	24.5 (19.0–50.5)	11.0 (3.0–22.0)	<0.001
Total count (x10^6^)	151.1 (86.5–540.0)	45.6 (25.0–302.0)	<0.001
Sperm DFI (%)^a^	19.9 (15.6–24.4)	29.9 (26.6–34.3)	<0.001

*Vol* semen volume, *Con* sperm concentration, *PR* progressive motility, *NP* non-progressive motility, *Morph* morphology

^a^ Numeric data with abnormal distribution were presented by median (Q1-Q3) value

**Fig. 1:**
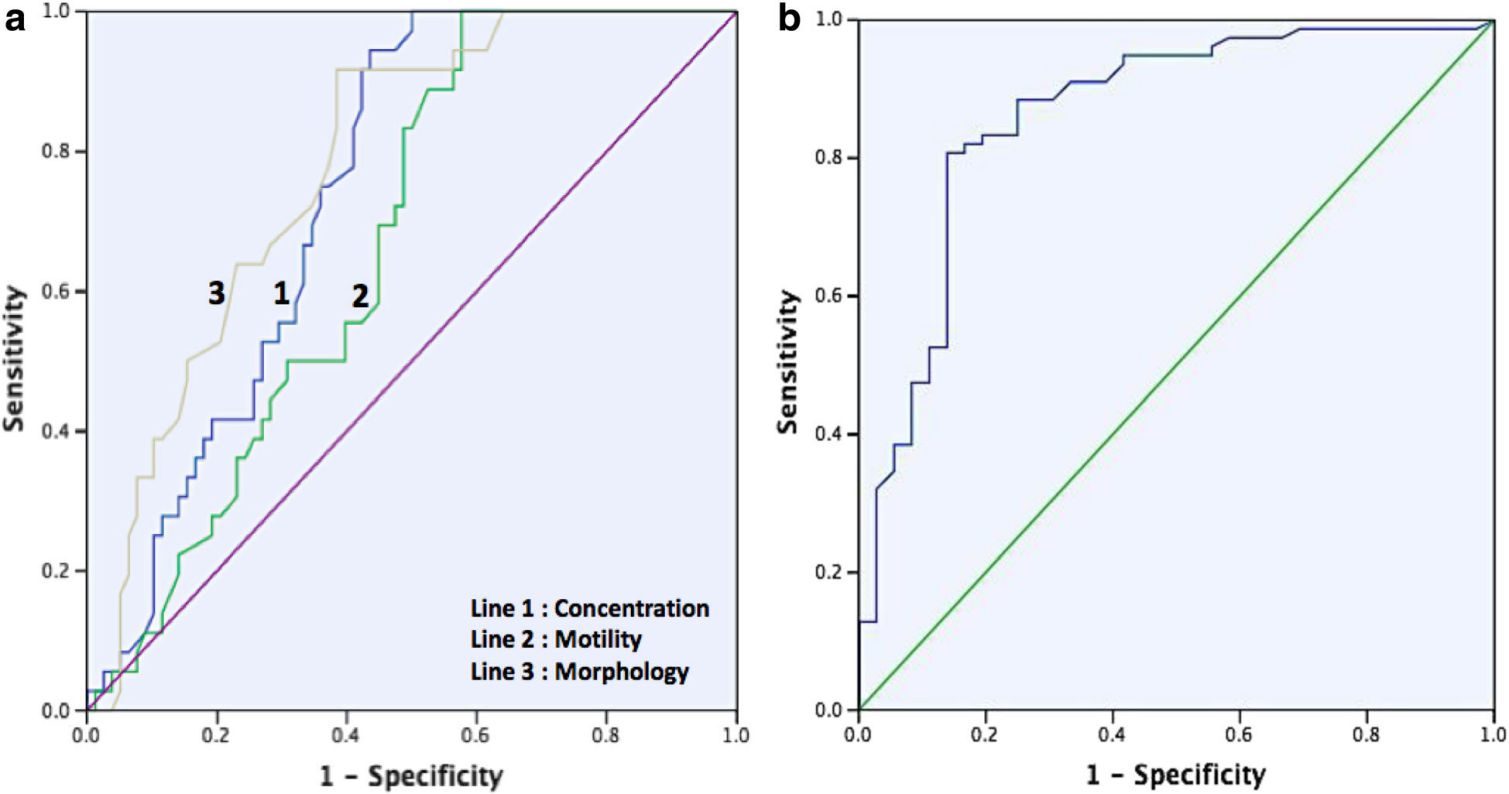
ROC curve of **a** semen analysis compared to **b** sperm DFI to diagnose male infertility. The three semen parameters: 1) concentration, 2) motility, and 3) morphology have lower AUC compared to sperm DFI

The ability of sperm DFI and semen analysis (concentration, motility, and morphology) to diagnose male infertility was described by ROC curve (Fig. 1). AUC of sperm DFI was 0.862 (95% CI 0.783, 0.941), which was higher than concentration (AUC of 0.744; 95% CI 0.657, 0.831), motility (AUC of 0.668; 95% CI 0.572, 0.765), and morphology (AUC of 0.718; 95% CI 0.697, 0.864) of semen analysis (Table 2).

**Table 2:** AUC comparison of sperm DFI and semen parameters according to the 36 fertile and 78 infertile men

	AUC	p	95%CI
DFI	0,862	<0,001	(0,783–0,941)
Con	0,744	<0,001	(0,657–0,831)
Mot	0,668	<0,05	(0,572–0,765)
Morph	0,718	<0,001	(0,697–0,864)

*DFI* DNA fragmentation index, *Con* sperm concentration, *Mot* sperm motility, *Morph* sperm morphology, *AUC* area under curve, *CI* confidence interval

The highest sensitivity and specificity for sperm DFI was found at the cut-off point of 26.1%. With this cut-off point, sperm DFI was able to distinguish infertile men from fertile men with sensitivity of 80.8% (95% CI; 70.0, 88.5), specificity of 86.1% (95% CI; 69.7, 94.8), positive predictive value of 92.6% (95% CI; 83.0, 97.3), negative predictive value of 67.4% (95% CI; 51.9, 80.0), positive likelihood ratio of 12.6 (95% CI; 5.4, 29.4), and negative likelihood ratio of 0.48 (95% CI 0.31, 0.75), as contained in Table 3. Logistic regression showed that sperm DFI of ≥26,1% has a prevalence ratio of 2.84 (95% CI; 1.86, 4.33) for infertility.

**Table 3:** Diagnostic value of Sperm DFI

	cut-off (%)	Sen (%)	Spec (%)	PPV (%)	NPV (%)	PLR	NLR	AUC	p	95% CI
Sperm DFI	26.1	80.8	86.1	92.6	67.4	12.6	0.48	0.862	<0.001	0.783–0.941

*sen* sensitivity, *spec* specificity, *PPV* positive predictive value, *NPV* negative predictive value, *PLR* positive likelihood ration, *NLR* negative likelihood ratio, *CI* confidence interval

## Discussion

The total number of subjects in the present study was relatively small, yet it fulfilled the minimal number required from the sample size calculation. From the demographic data, there was no significant difference in the age between the two groups. Aging is responsible for a general decrease in the function of tissues and organs, including the reproductive tissues and organs. The effects of aging on male fertility were studied by numerous studies. Robertshaw, et al., found that advanced paternal age has an adverse impact on ART outcomes [13]. Other study also found that the ability of spermatozoa to performed fertilization is inversely correlated with the age [14]. The effects of paternal age on fertility remain controversial. Wu, et al., found that increased paternal age had no impact on fertilization rate, embryo quality, and miscarriage rate after controlling the maternal age [15]. Ferreira, et al., who conducted a study to 1024 couples undergoing ICSI (intracytoplasmic sperm injection), also found no correlation between paternal age and sperm parameters or pregnancy rate [16]. Alfaraj and Yunus conducted a study to 451 couples and also found that advancing paternal age had no significant correlation with the outcomes of semen analysis parameters and IVF in infertile couples [17]. This study found no significant difference between age of the two groups. This might be caused by the subject’s selection method used in this study. The fertile group was taken from men proven to have living children and obtained from secondary infertile couples due to female factors so that the median and the range of the age were similar between the two groups.

We define the fertile men were those who have normal semen parameter (concentration, motility, and morphology), even though the subsequent subfertility condition might have correlated with the male factor that can not be seen in semen analysis result, such as body mass index (BMI). Although the data about male’s BMI were not provided, but it has been under a lot of suspicion as the cause of male infertility. There are emerging facts confirming that obesity negatively affects male reproductive potential not only by reducing sperm quality, but in particular, by altering the physical and molecular structures of germ cells in the testes, and ultimately by affecting the maturation and functions of sperm cells [18]. Anifandis, et al., found that BMI of men did not correlate with sperm parameters, but influenced the quality of the produced embryos which, in turns, influenced the pregnancy rates [19]. Similar result was also found by Petersen, et al., that showed couples with both partners having BMI > 25 kg/m^2^ had the lowest odds of live birth when compared to couples with both partners having BMI < 25 kg/m^2^ in IVF [20]. In contrast with those studies, Kupka, et al., retrospectively analyzed data retrieved from the National German IVF Registry, which covered 12 years and included 650,452 cycles, and found that the highest clinical pregnancy rates for both IVF and ICSI were seen in normal weight females with obese male partners (*P* = 0.0028) [21]. However, because none of those studies were randomized controlled trials, several potential confounders and biases might have influenced the findings.

This study found that sperm concentration, total motility, progressive motility, morphology, and total sperm count values were significantly lower in the infertile group compared to the fertile group, while semen volume did not differ. Although total sperm count, total motility, and progressive motility were significantly lower in infertile group, the median values were considered normal. It was considered that the normal median value of the total sperm count could be the result of the length of abstinence. Both groups have abstinence/delay of 7 days. The recommended abstinence delay for semen analysis are 2–7 days [12]. Many studies found that semen volume and concentration (resulted in total sperm count) increased with the increasing length of abstinence. Carlsen, et al., studied 419 semen samples with abstinence interval of 2–7 days and found that there was increased in semen volume and concentration after 4 days of abstinence and succeeding days, and there was no effect on motility and total motile spermatozoa with increased duration of abstinence [22]. Sunanda, et al., studied 730 men with abstinence interval of 2–7 days and found that semen volume and total count increased with the increasing abstinence period, while sperm motility and vitality declined after 5 days of abstinence [23]. The effects of abstinence length on sperm motility in previous studies were contradicted by a more recent study by Agarwal, et al., who conducted semen analysis by grouping the abstinence interval into three categories: short interval (1 day), the recommended interval of the World Health Organization (WHO) (2–7 days), and long interval (9–11 days). The study found significant increase in volume, total sperm count, total motility, and DNA fragmentation between the short and the recommended abstinence interval (*P* < .05); and between the recommended and the long abstinence interval (*P* < .05) [24].

In this study, the median value of sperm DFI was significantly higher in the infertile group compared to the fertile group (Table 1). The values of the three sperm parameters are also significantly lower in the infertile group. These results are consistent with the study of Sergerie, et al., in 2005 that found mean value of sperm DFI in the infertile group was significantly higher than in the fertile group (40.9 ± 14.3% compared to 13.1 ± 7.3%) and the mean sperm concentration in infertile group also significantly lower compared to the fertile group (62.9 ± 33.2 × 10^6^/ml compared to 102.4 ± 66.4 × 10^6^/ml) [25]. Similar result was also reported in a study that assessed the degree of DFI in patients dealing with infertility. Sperm DFI was significantly higher in patients with infertility compared to those of control (22.2 ± 5.6% vs. 16.7 ± 0.7%; *p* < 0.05) [26]. From these results, it may be concluded that sperm DFI determination could be used to distinguish infertile men from fertile men.

AUC value of sperm DFI was 0.862 (*p* < 0.001; 95% CI 0.783, 0.941) with sensitivity of 80.8% and specificity of 86.1%. Statistically, AUC value in the range of 80–90% has a good diagnostic strength [27]. This value is 11.8% higher than the highest AUC value of semen analysis that routinely performed in infertility workup (Table 2). This result indicates that clinically, sperm DFI has better diagnostic strength than semen analysis. Sperm DFI also has a stronger predictive value compared to free sperm DNA for the success of pregnancy in IVF and ICSI patients (AUC = 0.7; *p* < 0.05 compared to AUC = 0.6; *p* > 0.05) [28]. A meta-analysis by Cui, et al., also supports this result that the sperm DFI may be used to distinguish the sperm of infertile men from fertile men with AUC value of 0.921, and sensitivity of 80% and specificity of 83% [29].

The expected benefit of this study is to increase the outcome of infertility management in infertile couple population. Sperm DFI can well-distinguish infertile men from fertile men with positive predictive value of 92.6% at 26.1% cut-off point. Similar result was reported in a study comparing DNA fragmentation of neat and swim-up spermatozoa to predict pregnancy following ICSI. The cut-off value of the neat spermatozoa that resulted in 80% of pregnancy rate was 26% [30]. There are only a few published papers that specifically used SCD test (or Halosperm test) to assess male infertility and reported the correlation with IVF or ICSI outcomes. Two published papers reported that sperm DFI measured with Halosperm had no impact on the embryo quality and the ongoing pregnancy rates in IVF or ICSI. These studies, however, used different cut-off points from the present study (30 and 35% DFI) [31, 32]. Due to the higher cut-off points, the fact that extremely high DNA damages are associated with total pregnancy failure should not be ruled out. A new study that uses 26% DFI as a cut-off point is needed to establish the impact of sperm DFI measured with Halosperm on male infertility.

In this study, the prevalence ratio for sperm DFI ≥ 26.1% was 2.84 (95% CI, 1.86, 4.33). Thus, it may be concluded that a man with sperm DFI of ≥ 26.1% has 2.84 times greater risk for infertility than men with sperm DFI of < 26.1%. These results are consistent with a cohort study that found the most predictive cut-off point for pregnancy was sperm DFI of > 25.5% with negative predictive value of 72.7% and the odds ratio for sperm DFI < 25.5% was 3.6 (95% CI; 1.66, 7.82) [33].

## Conclusion

This study discovered a significant difference between the median value of sperm DFI of infertile and fertile men. In determining male infertility, the diagnostic value (AUC) of sperm DFI was higher than the semen analysis. Sperm DFI of 26,1% was the optimal cut-off point to distinguish infertile men from fertile men with sensitivity of 80.8%, specificity of 86.1%, positive predictive value of 92.6%, and negative predictive value of 67.4%. Thus, the determination of sperm DFI can be considered as an additional diagnostic tool besides the semen analysis before an infertile couple undergoing an ART. In addition, in order to decide whether the cut-off point of 26.1% can be used universally for sperm DFI determination by SCD method using Halosperm® Kit, it is necessary to perform further research in a large scale.

## Abbreviations

AUC: Area under the curve
CI: Confidence interval
DFI: DNA fragmentation index
DNA: Deoxyribonucleic acid
ICSI: Intra cytoplasmic sperm injection
IVF: In vitro fertilization
NLR: Negative likelihood ratio
NPV: Negative predictive value
PLR: Positive likelihood ratio
PPV: Positive predictive value
ROC: Receiver operator characteristic
SCD: Sperm chromatin dispersion test
WHO: World Health Organization
